# Inhibitory effect of roburic acid in combination with docetaxel on human prostate cancer cells

**DOI:** 10.1080/14756366.2021.2018684

**Published:** 2022-01-05

**Authors:** Xiao Wang, Xu Xuetao, Mengshuo Wu, Panpan Wu, Zhaojun Sheng, Wenfeng Liu, Yan-Yan Ma, Den-Gao Zhao, Kun Zhang, Dongli Li, Xi Zheng, Susan Goodin

**Affiliations:** a School of Biotechnology and Health Sciences, Wuyi University, Jiangmen City, China; b International Healthcare Innovation Institute (Jiangmen), Jiangmen City, Guangdong Province, China; c Department of Chemical Biology, Ernest Mario School of Pharmacy, Rutgers, The State University of New Jersey, Piscataway, NJ, USA; d Rutgers Cancer Institute of New Jersey, New Brunswick, NJ, USA

**Keywords:** Prostate cancer, roburic acid, docetaxel, combination

## Abstract

Roburic acid (ROB) is a naturally occurred tetracyclic triterpenoid, and the anticancer activity of this compound has not been reported. Docetaxel (DOC) is the first-line chemotherapeutic agent for advanced stage prostate cancer but toxic side effects and drug resistance limit its clinical success. In this study, the potential synergistic anticancer effect and the underlying mechanisms of ROB in combination with DOC on prostate cancer were investigated. The results showed that ROB and DOC in combination synergistically inhibited the growth of prostate cancer cells. The combination also strongly induced apoptosis, and suppressed cell migration, invasion and sphere formation. Mechanistic study showed that the combined effects of ROB and DOC on prostate cancer cells were associated with inhibition of NF-κB activation, down regulation of Bcl-2 and up regulation of Bax. Knockdown of NF-κB by small interfering RNA (siRNA) significantly decreased the combined effect of ROB and DOC. Moreover, we found that esomeprazole (ESOM), a proton pump inhibitor (PPI), strongly enhanced the effectiveness of ROB and DOC on prostate cancer cells in acidic culture medium. Since acidic micro environment is known to impair the efficacy of current anticancer therapies, ESOM combined with ROB and DOC may be an effective approach for improving the treatment of prostate cancer patients.

## Graphical abstract

**Figure:**
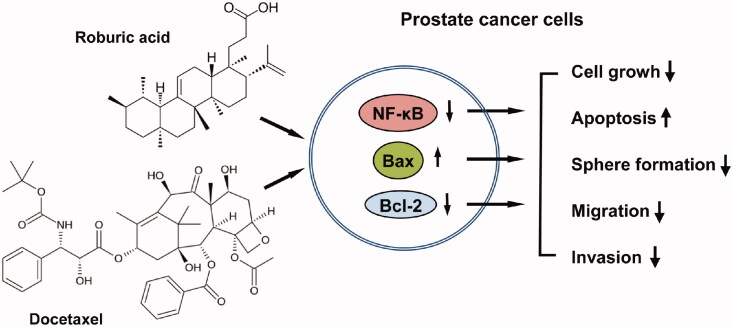

## 1. Introduction

Prostate cancer is the most prevalent male urogenital malignancy and the second leading cause of cancer death in US men^1^. Risk factors including advancing age, race, geographical distribution, diet and family history are contributing to the incidence of this disease^2^^,^^3^. For the treatment options of prostate cancer, early stages of the disease can be managed with active surveillance, radical prostatectomy, or radiation therapy. Patients who are not suitable for surgical intervention can be treated with androgen deprivation^4^. However, prostate cancer cells lose their hormone dependence and therapeutic responsiveness during disease progression^5^^,^^6^. When the disease progresses to the hormone-refractory stage (also referred to as androgen-independent), chemotherapy is the only option left for these patients^7^^,^^8^.

**Figure 1.**
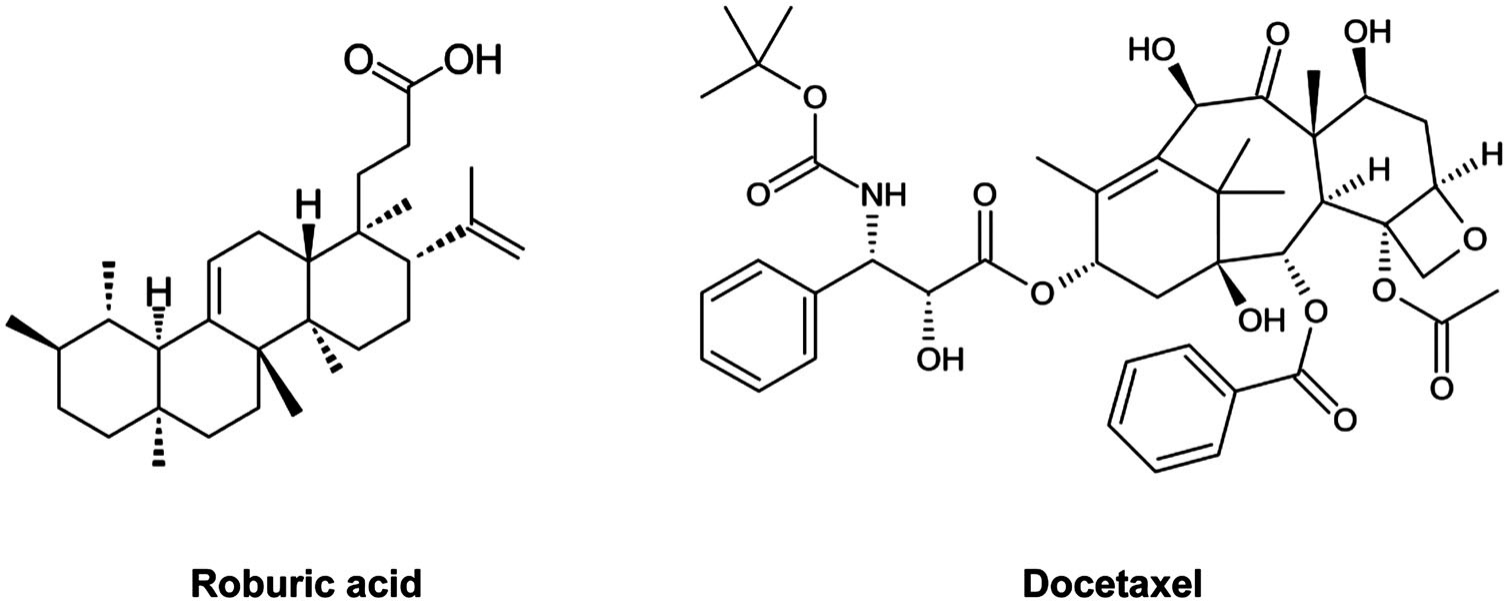
Structures of roburic acid (ROB) and docetaxel (DOC).

Docetaxel (DOC; Figure 1) is an anti-mitotic agent, which is able to restrain microtubule disassembly and prevent the formation of mitotic spindles. DOC chronically activates the spindle assembly checkpoint (SAC), which in turn leads to mitotic arrest and eventually induces cell death^9^^,^^10^. DOC is the first-line chemotherapy for treatment of advanced stage prostate cancer^11^^,^^12^. The combination of DOC with androgen deprivation therapy was also used in metastatic or high-risk localised hormone-sensitive prostate cancer^13^. The survival of patients after DOC chemotherapy remains limited. Systemic side effects of DOC hamper the patient’s compliance and drug resistance invariably emerges, leading to disease relapse. Therefore, approaches to improve DOC-based chemotherapy in prostate cancer patients are urgently needed.

It is of highly clinical significance to identify agents that have synergistic effect when combined with DOC. Synergistic combinations may allow to decrease the dose of DOC without reducing its effectiveness. Lowering the dose of DOC will decrease its toxic side effects. Effective combinations may also overcome DOC resistance. Roburic acid (ROB; Figure 1) is a naturally occurred tetracyclic triterpenoid. ROB was originally isolated from oak galls, and later also found in Gentiana macrophylla Pall, etc.^14–16^. The bioactivity of ROB was rarely reported. A study conducted by Chen et al. demonstrated that ROB inhibited nitric oxide (NO) and interleukin (IL)-6 via targeting nuclear factor kappa B (NF-κB) and mitogen-activated protein kinase (MAPK) pathway in murine macrophage RAW264.7 cells^17^. However, the anticancer activity of ROB has not been reported.

It is well known that the Warburg effect leads tumours to become acidic, and the acidic extracellular pH is a well recognised feature of tumour environment^18^. The tumour microenvironment acidification is a real phenotype that bursts the malignant behaviour of virtually all tumours, including prostate cancer^19^. Acidic tumour microenvironment increases tumour invasion, metastasis and chemotherapeutic drug resistance^19^. Interestingly, tissue acidification is common to type II diabetes as well^20^, suggesting that microenvironmental acidity is a common pathway of many human pathologies. Therefore, it is important to determine the activities of potential new anticancer compounds such as roburic acid in buffered pH and low pH conditions. Moreover, many laboratory-based and clinical studies have demonstrated that tumour acidity decreased the efficacy of chemotherapeutic agents, and antiacidic drugs such as proton pump inhibitors (PPIs) improved the effectiveness of anticancer therapies^21–34^. Combined potential new anticancer agents with PPIs is highly relevant for developing effective anticancer combination therapies.

In the present study, we investigated the inhibitory effects of ROB, DOC and ESOM alone or in combination on human prostate cancer cells in pH7.4 and pH6.5 culture conditions. The mechanisms associated with the effects of ROB in combination with DOC was determined. Results of the present study demonstrated for the first time that ROB combined with DOC had synergistic effect on inhibiting the growth of prostate cancer cells. The combination also induced apoptosis, suppressed cell migration, invasion and sphere formation. Mechanistic studies indicate that the effects of ROB and DOC were associated with inhibition of NF-κB activation, down regulation of Bcl-2 and up regulation of Bax. Furthermore, we found that ESOM strongly enhanced the efficacy of ROB and DOC on prostate cancer cells in acidic culture medium. The strong combined effect of ROB, DOC and ESOM warrants further in vivo studies using suitable animal models.

## 2. Materials and methods

### 2.1. Cell line and cell culture

Human prostate cancer cell lines (LNCaP, VCaP and PC-3) were purchased from the American Type Culture Collection (ATCC, Rockville, MD, USA). The cells were propagated in Roswell Park Memorial Institute (RPMI) medium supplemented with 10% foetal bovine serum (FBS), penicillin (100 units/ml), streptomycin (100 mg/ml), and L-glutamine (300 µg/ml) (All from Gibco, Grand Island, NY, USA). The acidic cell culture medium (pH 6.5) was obtained by the addition of 1 M HCl solution. The cells were grown at 37 °C in a humidified atmosphere of 5% CO_2_. ESOM, ROB and DOC were obtained from Sigma-Aldrich (St. Luis, MO, USA). The compounds were dissolved in DMSO to make stock solution, and the final concentration of DMSO in all experiments was 0.2%.

### 2.2. Cell viability assay

The cells were seeded at a density of 1.5 × 10^4^ cells/ml of medium in a 96-well plate (0.2 ml/well). The cells were incubated for 24 h before treatment with DOC and/or ROB for 72 h. Following treatment, 10 µL Cell Counting Kit-8 (CCK-8) (CK04-11, VitaScientific, Beltsville, MD, USA) was added into each well and the cells were incubated for 30 min. A microplate reader ChroMate™ (Awareness Technology, Palm City, FL, USA) was used to detect the optical density (OD) at a wavelength of 450 nm.

### 2.3. Apoptosis assay

Apoptosis was determined by morphological assessment in cells stained with propidium iodide (PI). After each experiment, cells were trypsinized and cytospin slides were prepared. The cells were fixed with acetone/methanol (1:1) for 10 min at room temperature, followed by 10 min with propidium iodide staining (1 µg/ml in PBS), and finally analysed using the CELENA® fluorescence imaging system (Lagos Biosystem, Annandale, VA, USA).

### 2.4. Caspase-3 assay

Caspase-3 activation was measured using an EnzoLyte AMC Caspase-3 Assay Fluorimetric kit (AnaSpec, Fremont, CA) following the manufacturer instructions. Briefly, 1 × 10^5^ cells were plated in triplicate in a flat-bottomed 96-well plate. After drug treatment, caspase-3 substrate was added to each well. Plates were incubated for 30 min at room temperature. Fluorescence intensity was measured in a Tecan Inifinite M200 plate reader (Tecan US Inc., Durham NC, USA). For the caspase-3 immunofluorescence staining, cytospin of PC-3 cells were fixed in acetone/methanol (1:1) for 10 min at room temperature. The cells were incubated with caspase-3 primary antibody (active form, #9661, Cell Signalling Tech, Danvers, MA, USA). After washing with PBS, the cells were incubated with FITC-conjugated secondary antibody. The caspase-3 immunofluorescence staining was examined using the CELENA® fluorescence imaging system (Lagos Biosystem, Annandale, VA, USA).

### 2.5. Sphere formation assay

PC-3 cells were seeded at a density of (1 × 10^3^ cells/mL) in ultralow attachment surface culture plate (Corning Co, NY, USA) and cultured in Keratinocyte SFM medium supplemented with EGF, insulin, selenium and transferrin (Gibco, Grand Island, NY, USA). The cells were treated with ROB and/or DOC. On day 12, the numbers of spheres were counted under an inverted microscope (Optiphot, Nikon, Tokyo, Japan).

### 2.6. Cell migration assay

PC-3 cells were seeded in 6-well culture plates at a density of 8 × 10^4^ cells/ml and grown to confluency. Monolayers of confluent PC-3 cells were then wounded using a 200 μl pipette and washed with PBS. The cells were treated with ROB and/or DOC, and cell migration was monitored using an inverted microscope (Optiphot, Nikon, Tokyo, Japan), and images were obtained at 0 h and 24 h. The wounded areas were measured by ImageJ software and the relative wound closure was expressed as a ratio of the original scratch area.

### 2.7. Invasion assays

PC-3 cells (2 × 10^4^ cells/well) were plated in serum-free RPMI medium in the top chamber with a membrane (24 well insert; pore size, 8 µm; Corning Inc.) coated in Matrigel (1 mg/ml; BD Biosciences, San Jose, CA, USA). RPMI medium containing 10% serum was used as a chemoattractant in the lower chamber. Following incubation for 24 h, a cotton swab was used to remove the non-migrated cells in the upper chamber and the filters were individually stained with 2% crystal violet. The migrated cells adhering to the underside of the filter were examined and counted under a light microscope (Optiphot, Nikon, Tokyo, Japan).

### 2.8. Nf-κB-dependent reporter gene expression assay

PC-3/N cells^35^ were treated with ROB and/or DOC for 24 h, and then the cells were harvested in 1× reporter lysis buffer (Promega, Madison, WI, USA). After centrifugation, 10 µl aliquots of the supernatants were mixed with 10 µl of luciferase substrate (Promega) and measured for the luciferase activity by using a Luminometer LuMate™ (Awareness Technology, Palm City, FL, USA). The luciferase activity was normalised against known protein concentrations and expressed as the percentage of luciferase activity in the control cells. The protein level was determined by Bio-Rad protein assay kits (Bio-Rad, Hercules, CA, USA) according to the manufacturer's instructions.

### 2.9. Transfection with NF-κB p65 siRNA

NF-κB p65 small interfering RNA (siRNA) and scrambled negative control siRNA were from Cell Signalling Tech (Danvers, MA, USA). PC-3 cells were seeded in 6-well plates and incubated for 24 h to allow adherence of cells. SiRNA was transfected into the cells using Lipofectamine 2000 (Thermo Fisher Scientific) according to the manufacturer’s instruction. The final concentration of siRNA was 100 nM. After 48 h, the cells were harvested for expression assay or treating with ROB and/or DOC.

### 2.10. Western blotting

After treatment, the cells were lysed and the proteins were subjected to sodium dodecyl sulfatepolyacrylamide gel electrophoresis (SDS-PAGE) and transferred to nitrocellulose membranes. After blocking non-specific binding sites with blocking buffer, the membranes were incubated overnight at 4 °C with primary antibodies (#6956 for NF-κB p65, #15071 for Bcl-2, #5023 for Bax and #2808 for survivin; Cell Signalling Tech, Beverly, MA, USA). β-actin (sc-47778, Santa Cruz Biotechnology Inc., Dallas, TX, USA) was used as a loading control. Following the removal of the primary antibodies, the membranes were washed three times with TBS (PBS containing 0.05% Tween 20) buffer at room temperature and later incubated with fluorochrome-conjugated secondary antibody (925–32211, Li-Cor Biotechnology, Lincoln, NE, USA). The membrane was then washed with TBS three times. Final detection was done with an Odyssey infra-red imaging system (Li-Cor Biotechnology).

### 2.11. Measurement of intracellular pH change

The change in intracellular pH was measured as previously described^22^. Briefly, cells grown on 35 mm dishes were treated with ESOM for 24 h and incubated with 1 µg/mL BCECF-AM solution for 30 min. Fluorescence staining was determined using using the CELENA® fluorescence imaging system.

### 2.12. Statistical analyses

Statistical analyses were done by using the software InStat (GraphPad Software, Inc., La Jolla, CA, USA). Comparisons of treatment outcome were analysed for statistical difference by ANOVA. Statistical significance was assumed at a value of *p* < 0.05. Analysis of synergy was performed using the CompuSyn software based on the isobologram priciple. The combination index (CI) was used for data analysis of two-drug combinations. Index values of CI < 1, CI = 1 and CI > 1 indicate synergism, additive effect and antagonism, respectively.

## 3. Results and discussion

### 3.1. Effects of ROB on human prostate cancer cells and normal prostate epithelial cells

**Figure 2.**
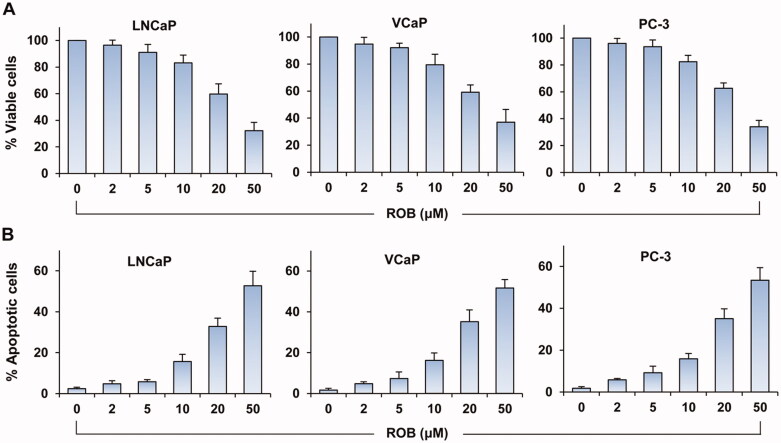
Effects of ROB on growth and apoptosis in human prostate cancer cells. LNCaP, VCaP and PC-3 cells were treated with different concentrations of ROB for 72 h. The cell viability was determined by CCK-8 assay and apoptosis was examined by PI staining. (A) Percentage of cell viability and (B) percent apoptosis after treatment with ROB. Each value is the mean ± SD from three separate experiments.

**Figure 3.**
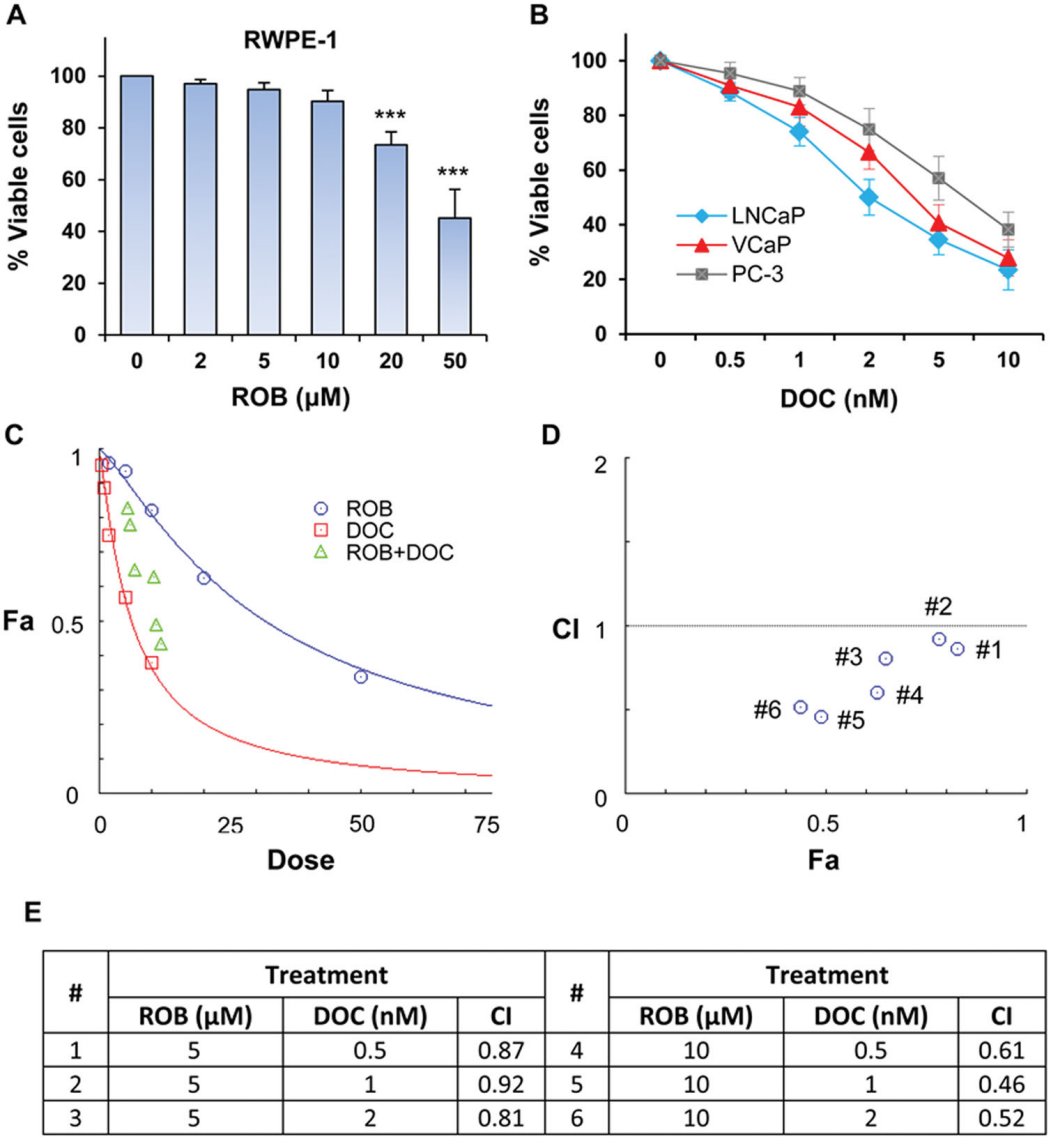
Effect of ROB on RWPE-1 cells, and effects of DOC and ROB on prostate cancer cells. (A) RWPE-1 cells were treated with different concentrations of ROB for 72 h and the cell viabililty was determined by CCK-8 assay. (B) LNCaP, VCaP, and PC-3 cells were treated with different concentrations of DOC for 72 h and the cell viabililty was determined by CCK-8 assay. (C) PC-3 cells were treated with ROB and DOC alone or in combination. Effect of the treatments on cell viability was plotted as fraction affected (Fa). (D, E) Combination index (CI) for different dose ratios of DOC and ROB in combinations. ***Indicates significant differences (*p* < 0.001) as compared to the control.

In initial studies, we determined the effects of ROB on cell growth and apoptosis in human prostate cancer LNCaP, VCaP and PC-3 cells. After treatment with ROB for 72 h, all three cell lines exhibited dose-dependent decreases in cell viability (Figure 2(A)). Treatment of the cells with ROB resulted in apoptosis in a dose-dependent manner (Figure 2(B)). ROB had similar effects on cell viability and apoptosis among the three prostate cancer cell lines tested. We also determined the effect of ROB on cell viability of normal prostate epithelial RWPE-1 cells. As shown in Figure 3(A), ROB at low doses did not affect the cell viability while at doses of 20 and 50 μM, significant decreases in cell viability were seen in the cells (Figure 3(A)).

### 3.2. Inhibitory effect of ROB and DOC in combination on human prostate cancer cells

To explore the potential synergistic effect of ROB and DOC, we first determined the inhibitory effect of DOC on prostate cancer cells. As shown in Figure 3(B), treatment LNCaP, VCaP and PC-3 cells with DOC dose-dependently decreased the cell viability. Since DOC is clinically used in late stage prostate cancer patients, the androgen-independent PC-3 cells were used in subsequent experiments to determine the combined effect of ROB and DOC. PC-3 cells were treated with ROB at non-toxic doses in combination with low doses of DOC, and the potential synergistic effect of the combinations was determined using the CompuSyn software. As shown in Figures 3(C,D), combinations of ROB and DOC at different dose ratios had synergistic effect. The CI for each combination of ROB and DOC is shown in Figure 3(E). ROB (10 µM) combined with DOC (1 nM) had the lowest CI (0.46) among the combinations indicating a strong synergistic effect.

### 3.3. Effect of ROB and DOC in combination on apoptosis of PC-3 cells

**Figure 4.**
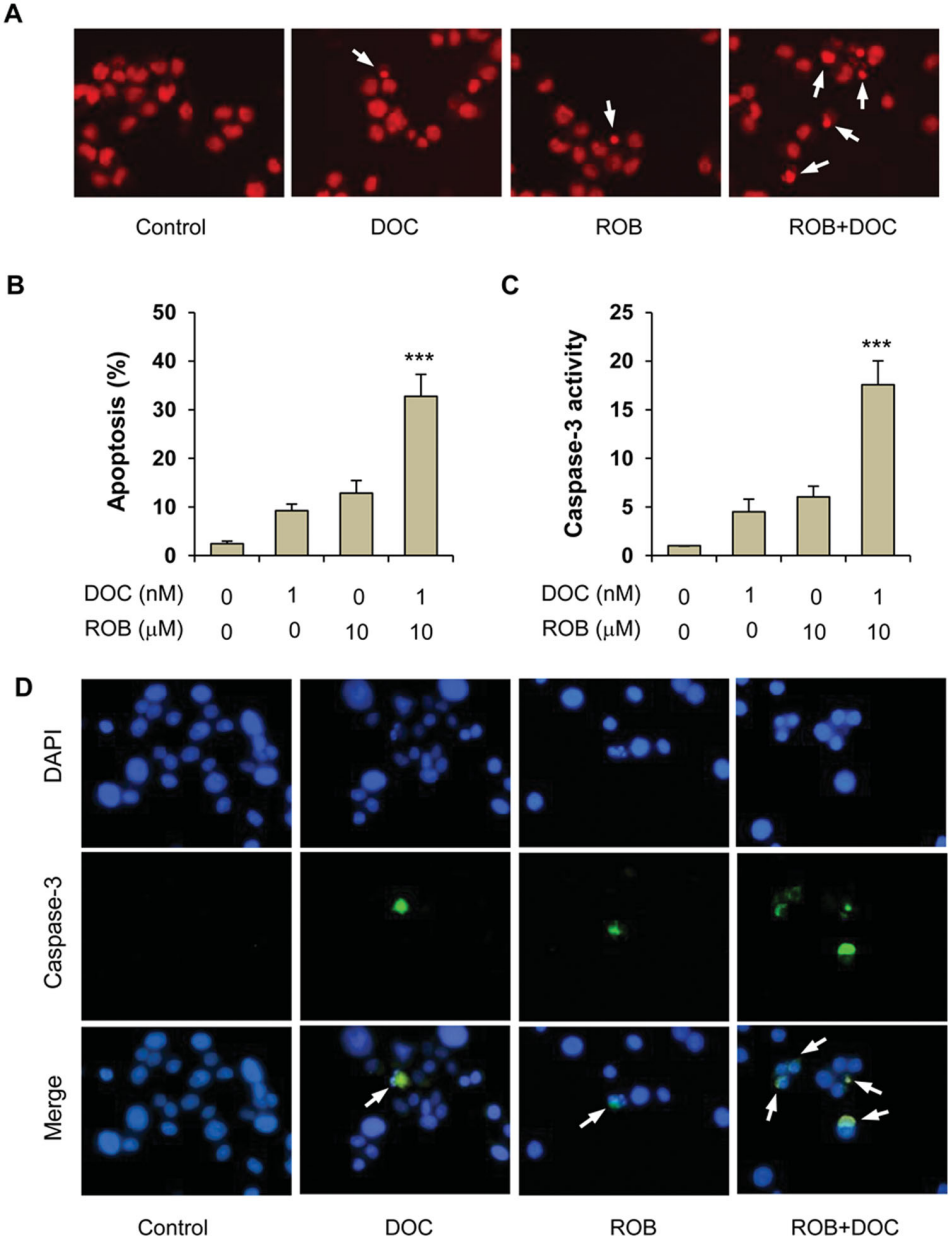
Apoptotic effect of ROB and DOC alone or in combination on PC-3 cells. PC-3 cells were treated with ROB and/or DOC for 72 h and apoptosis was determined by PI staining and caspase-3 assays. (A) Representative micrographs of PI staining in PC-3 cells treated with ROB and/or DOC. (B) Percent apoptotic cells as measured by PI staining. (C) Caspase-3 activity in PC-3 cells treated with ROB and/or DOC. (D) Representative micrographs of caspase-3 (cleaved form) immunofluorescence in PC-3 cells treated with ROB and/or DOC. Each value represents mean ± S.D from three separate experiments. ***Indicates significant differences (*p* < 0.001) as compared to cells treated with ROB or DOC alone.

The effects of ROB and DOC alone or in combination on apoptosis of PC-3 cells were assessed by propidium iodide staining and caspase-3 assays. It was observed in PI staining assay that DOC and ROB alone had small to moderate effects on stimulating apoptosis, while the combination of these two agents strongly induced apoptosis. Representative micrographs of PI staining in the untreated, ROB-treated, DOC-treated and combination-treated cells are shown (Figure 4(A)). As shown in Figure 4(B), cells treated with the combination of ROB and DOC had significantly more apoptotic cells than that in cells treated with ROB or DOC alone (*p* < 0.001). The effect of ROB and DOC on apoptosis was confirmed by the caspase-3 assay. As shown in Figure 4(C), caspase-3 activity was strongly increased in the cells treated with the combination of ROB and DOC (*p* < 0.001 as compared to DOC- or ROB-treated cells). Figure 4(D) shows representative micrographs of caspase-3 (active form) immunofluorescence staining in untreated, DOC-treated, ROB-treated and DOC + ROB-treated cells.

### 3.4. Inhibitory effect of ROB and DOC on PC-3 cell migration and invasion

**Figure 5.**
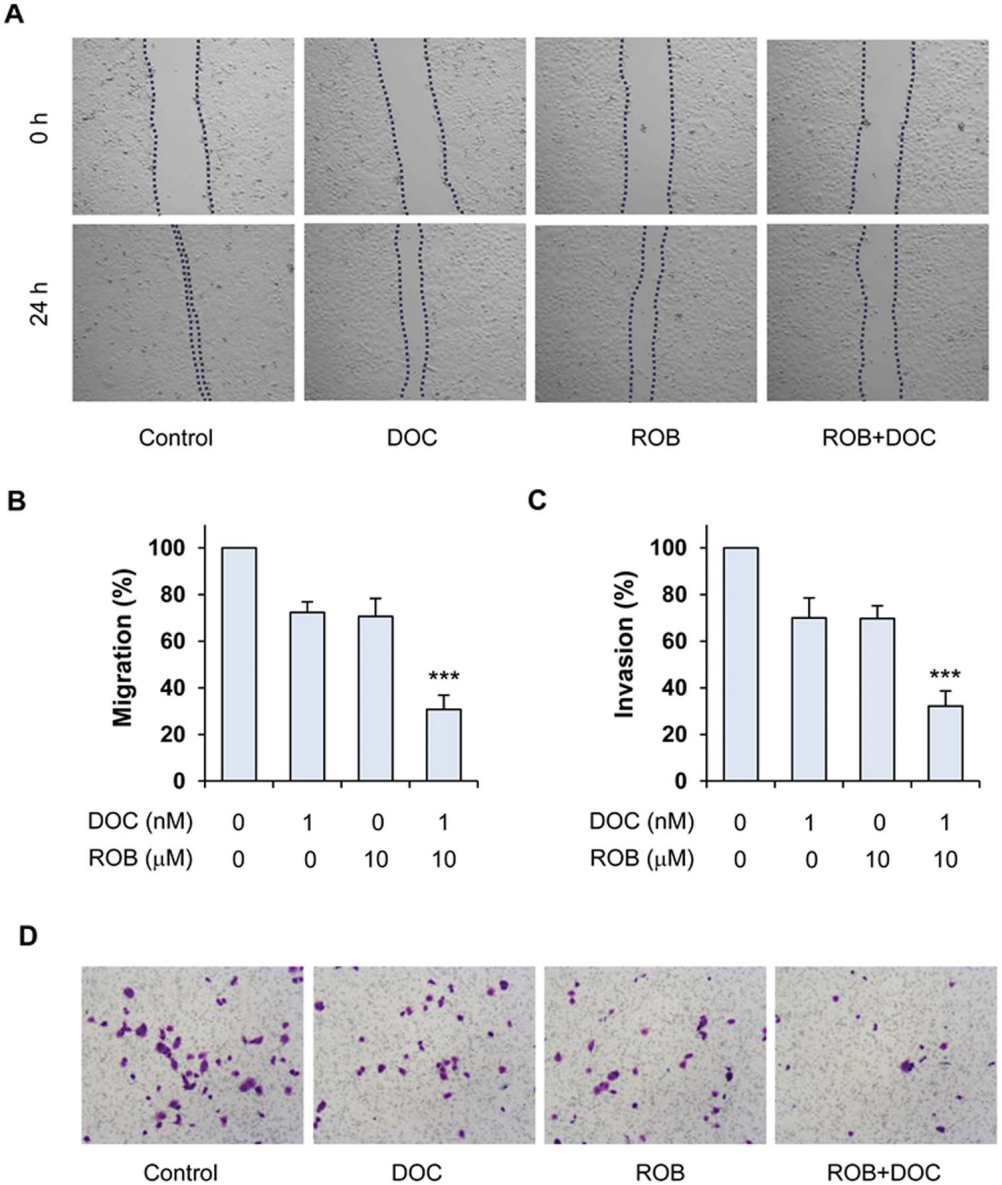
Effects of ROB and DOC alone or in combination on migration and invasion of PC-3 cells. For the migration assay, PC-3 cells were wounded followed by treatment with ROB and DOC alone or in combination for 24 h. (A) Representative micrographs taken at 0 and 24 h. (B) Cell migration rate as determined by Image J in cells treated with ROB and/or DOC. For the invasion assay, PC-3 were seeded in transwell coated with matrigel and treated with ROB and/or DOC for 24 h. (C) Number of invasive cells in PC-3 cells treated with ROB and DOC alone or in combination. (D) Representative micrographs of invasive cells on the transwell membrane. Each value represents mean ± S.D from three separate experiments. ***Indicates significant differences (*p* < 0.001) as compared to cells treated with ROB or DOC alone.

We next determined the effects of DOC and ROB on migration and invasion of PC-3 cells. Scratch wound healing assay was used to examine the migration of PC-3 cells. Following incubation with DOC and ROB for 24 h, the migration of PC‑3 cells to the denuded area was inhibited (Figure 5(A)). The combination of ROB and DOC had stronger effect than either agent alone on suppressing the migration of PC‑3 cells. Statistical analysis using ANOVA showed that the migration rate in the combination-treated group was significantly lower than that in ROB- or DOC-treated group (*p* < 0.001). Results of the invasion assay revealed that ROB and DOC inhibited the invasion of PC‑3 cells through the Matrigel-coated filter pores (Figure 5(B)). The combination of DOC and ROB strongly inhibited cell invasion. Statistical analysis using ANOVA showed that the migration rate in the combination-treated group was significantly lower than that in ROB- or DOC-treated group (*p*< 0.001). Taken together, the results described above revealed that DOC and ROB in combination strongly suppressed cellular migration and invasion of PC‑3 cells.

### 3.5. Suppression of sphere formation in prostate cancer cells by ROB and DOC

**Figure 6.**
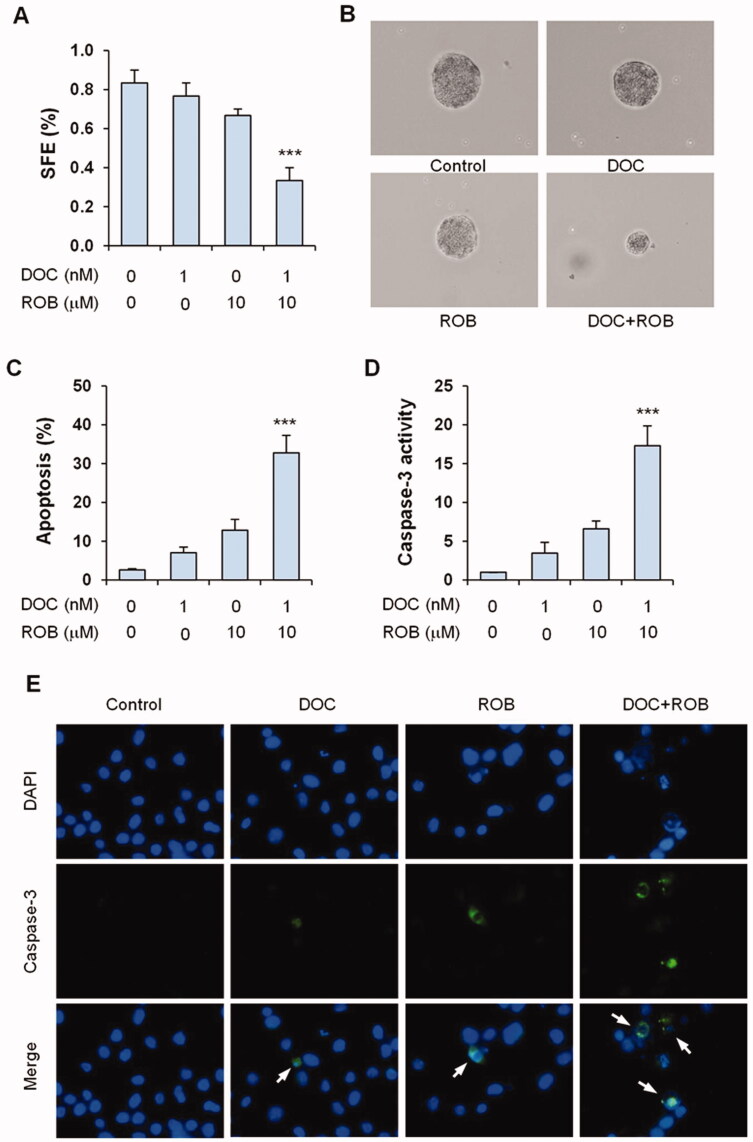
Effects of ROB and DOC on sphere formation and apoptosis of sphere-forming cells. For the sphere formation assay, PC-3 cells were cultured in ultralow attachment plate with serum-free medium and treated with ROB and/or DOC for 12 days. The number of spheres was counted under a microscope. (A) Sphere forming efficiency (SFE) in PC-3 cells treated with ROB and/or DOC. (B) Representative micrographs of spheres from cells treated with ROB and DOC alone or in combination. For the apoptosis assay, PC-3 spheres formed in serum-free medium were dissociated by Accutase® and treated with ROB and/DOC for 72 h. (C) Apoptosis as determined by PI staining. (D) Caspase-3 activity. (E) Representative micrographs of caspase-3 (cleaved form) immunofluorescence staining in PC-3 cells treated with ROB and/or DOC. Each value represents mean ± S.D from three separate experiments. ***Indicates significant differences (*p* < 0.001) as compared to cells treated with ROB or DOC alone.

Cancer stem cells (CSCs) are a small sub-population of tumour cells that retain the ability to renew and differentiate^36^. CSCs have self-renewal capacity and are considered to play important roles in cancer chemoresistance and relapse^37^^,^^38^. It is well characterised that CSCs can form three dimensional spheres *in vitro* in serum-free suspension cultures^39^^,^^40^. Therefore, we investigated the effects of ROB and DOC on PC-3 cell sphere formation in serum free medium. When culturing in ultralow attachment plate with serum-free medium, a small fraction of the PC-3 cells formed spheres. Treatment with DOC had little effect on sphere forming efficiency (SFE) and ROB alone had a moderate effect on decreasing SFE (Figure 6(A)). The combination of ROB and DOC strongly decreased the SFE in PC-3 cells (*p* < 0.001 as compared to ROB- or DOC-treated group). Treatment with ROB combined with DOC also decreased the size of the spheres (Figure 6(B)).

We also investigated if ROB and DOC induced apoptosis in PC-3 sphere-forming cells. The spheres formed in serum-free culture medium were dissociated by Accutase® to make single cell suspension, and the cells were treated with DOC or ROB alone or in combination. As shown in Figure 6(C), treatment with DOC or ROB alone had small to moderate effect on increasing apoptosis while the combination had a significantly stronger effect on apoptosis than either agent used alone (*p* < 0.001). Result of the caspase-3 activity assay and immunofluorescence staining confirmed that DOC combined with ROB had potent effect on increasing apoptosis in sphere-forming cells (Figure 6(D)). Representative micrographs of caspase-3 (active form) immunofluorescence staining in the cells treated with DOC and ROB alone or in combination are shown in Figure 6(E). Since the sphere formation assay is a classic method for the analysis of self-renewal ability^41^, our result indicates that the combination of ROB and DOC suppresses the self-renewal of prostate CSCs. Moreover, the strong apoptotic effect of the ROB and DOC combination on sphere-forming cells suggests that DOC combined with suitable agents may provide an effective approach for eliminating prostate CSCs.

### 3.6. Effects of ROB and DOC on NF-κB, Bcl-2, Bax and survivin in PC-3 cells

**Figure 7.**
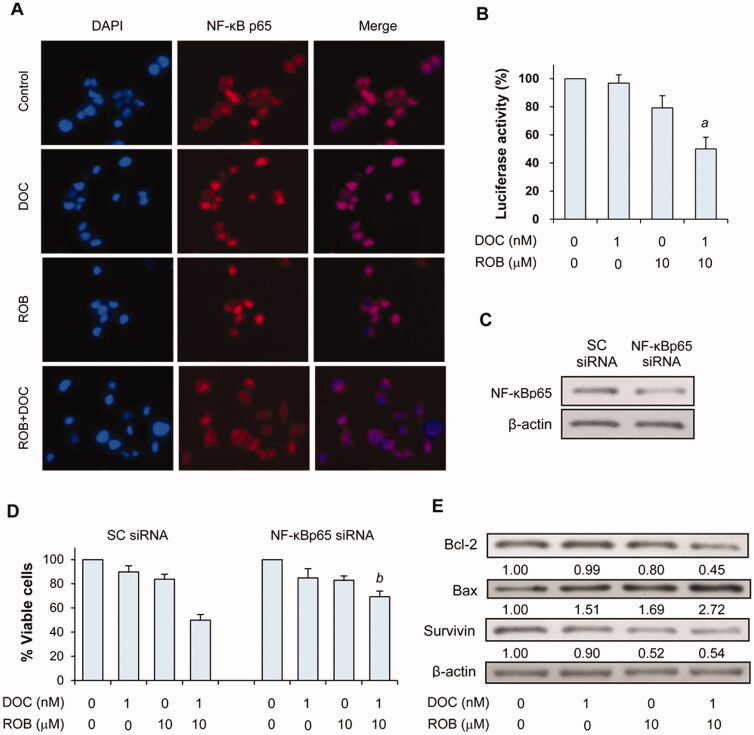
Effects of ROB and DOC on NF-κB, Bcl-2, Bax and survivin in PC-3 cells. (A) Cellular localiazation of NF-κB p65 protein in PC-3 cells treated with ROB and/or DOC for 24 h was examined by immunofluorescence staining. (B) NF-κB activity in PC-3/N cells was determined by the luciferase reporter assay. (C) Levels of Bcl-2, Bax and survivin in PC-3 cells treated with ROB and/or DOC was measured by Western blotting. (D) PC-3 cells were transfected with scrambled or NF-κB p65 siRNA, and the level of NF-κB p65 after transfection was determined by Western blotting. (E) PC-3 cells transfected with scrambled or NF-κB p65 siRNA were treated with ROB and/or DOC for 72 h. Cell viability was determined by CCK-8 assay. Data shown are mean ± SD from three experiments. *^a^*Indicates significant differences as compared to cells treated with DOC (*p* < 0.001) or ROB alone (*p* < 0.01). *^b^*Indicates significant differences (*p* < 0.01) as compared to the cells transfected with scrambled siRNA and treated with combination ROB and DOC.

To explore the mechanisms underlying the inhibitory effect of ROB and DOC on PC-3 cells, we investigated the effect of ROB and DOC on NF-κB activation in PC-3 cells. NF-κB is constitutively activated in human prostate cancer^42^, and previous studies have demonstrated that inhibition of NF-κB enhanced the efficacy of DOC in prostate cancer cells^43^^,^^44^. In our study, we first determined the cellular localisation of NF-κB by immunofluorescence. As shown in Figure 7(A), most of the untreated PC-3 cells showed nuclear staining of NF-κB. Cells treated with DOC also showed nuclear staining of NF-κB. ROB decreased nuclear staining in some cells while the combination of ROB and DOC strongly decreased the nuclear staining of NF-κB (Figure 7(A)). This result indicated that ROB + DOC strongly inhibited the nuclear translocation of NF-κB which is a critical step in the process of its activation^45^. To further evaluate if ROB and DOC suppressed the function of NF-κB, we used the luciferase reporter assay to determine the transcriptional activity of NF-κB. As shown in Figure 7(B), treatment of the cells with DOC had little effect on NF-κB transcriptional activity. ROB alone caused modest decrease in the transcriptional activity of NF-κB. Treatment with the combination of ROB and DOC strongly decreased the NF-κB transcriptional activity (Figure 7(B)). Statistical analysis using ANOVA demonstrated that NF-κB transcriptional activity was significantly lower in the combination-treated cells than in ROB- (*p* < 0.01) or DOC-treated cells (*p* < 0.001).

To further explore if the combined effect of ROB and DOC is mediated through NF-κB inhibition, we determined the influence of knocking down NF-κB through siRNA on the inhibitory effect of ROB and DOC. As shown in Figure 7(C), the level of NF-κB p65 protein was decreased in the cells transfected with NF-κBp65 siRNA. Knocking down NF-κB through siRNA significantly reduced the combined effect of ROB and DOC on decreasing the viability of PC-3 cells (*p* < 0.01, analysed by ANOVA). This result suggests that the inhibitory effect of ROB and DOC in combination is at least partially mediated through inhibition of NF-κB.

Since the combination of DOC and ROB induced apoptosis, we evaluated the influence of the combination treatment on the levels of Bcl-2 and Bax. As shown in Figure 7(E), treatment of PC-3 cells with the combination of DOC and ROB strongly decreased the level of Bcl-2 and increased the level of Bax. Proteins of the Bcl-2 family play a fundamental role in regulation of apoptosis^46^. Among members of the Bcl-2 family, Bcl-2 is a potent suppressor of apoptosis, whereas Bax is a pro-apoptotic protein. Higher levels of Bax relative to Bcl-2 may increase the susceptibility of cancer cells to apoptosis^47^. The result of our study suggests that ROB combined with DOC induced apoptosis in PC-3 cells through upregulation of Bax and downregulation of Bcl-2. We also determined the effect of ROB and DOC on the level of survivin, a member of the inhibitor of apoptosis (IAP) family which plays an important role in apoptosis regulation. However, no combined effect on decreasing the level of survivin was observed in cells treated with ROB and DOC in combination.

### 3.7. Effects of ROB and DOC on PC-3 cells in pH 7.4 and pH 6.5 culture media

**Figure 8.**
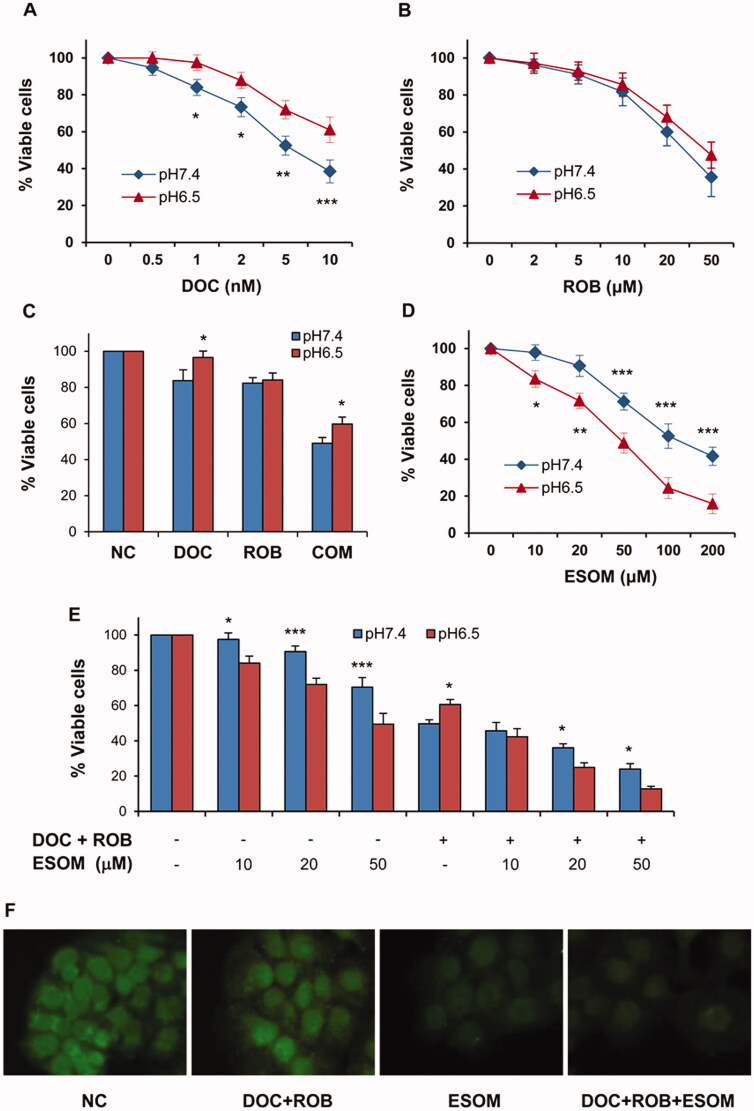
Influence of acidic culture medium and ESOM on the inhibitory effect of DOC and ROB on PC-3 cells. The cells were seeded in 96-well plate and treated with DOC or ROB alone or in combination (A–C), or treated with ESOM alone or together with DOC and ROB (D–E) in regular (pH7.4) or acidic (pH6.5) media. Cell viability was measured by the CCK-8 assay. Viability of PC-3 cells treated with DOC (A), ROB (B), DOC + ROB (C), ESOM (D), and ESOM together with DOC and ROB (E) is presented as percentage of the control. Each value represents mean ± S.D from three separate experiments. Asterisk indicates significant difference for cell viability between cells in pH7.4 and pH6.5 culture media (*p < 0.05, **p < 0.01, ***p < 0.001). For the intracellular pH mesurement, PC-3 cells treated with DOC (1 nM) + ROB (10 μM), ESOM (50 μM) or DOC (1 nM) + ROB (10 μM) + ESOM (50 μM) were incubated with BCECF-AM and the fluorescence was visualised using the CELENA® fluorescence imaging system.

PC-3 cells were cultured in pH7.0 medium and then in pH6.5 medium over a 3-week period to adapt the acidic culture condition. After that, the cells were treated with ROB and DOC alone or in combination in pH6.5 medium. For comparison, original PC-3 cells were cultured in normal medium (pH7.4) and treated with ROB and/or DOC. As shown in Figure 8(A), DOC induced a dose-dependent decrease in cell viability, and it was clear evident that PC-3 cells cultured in pH6.5 medium were more resistant to DOC than the cells cultured in pH7.4 medium (Figure 8(A)). This result is in line with a recent report showing that acidic pH-tolerant PC-3 cells were relatively resistant to DOC as compared to their parental cells (A). As shown in Figure 8(B), PC-3 cells cultured in acidic medium (pH6.5) were less sensitive to ROB as compared to PC-3 cells cultured in pH7.4 medium. However, the differences were not statistically significant (Figure 8(B)). As shown in Figure 8(C), PC-3 cells cultured in acidic medium (pH6.5) were also less sensitive to the combination of DOC and ROB. These results indicate that the acidic extracellular microenvironment is an important factor affecting the efficacy of anticancer agents. Potential new anticancer compounds should be tested for their effect on cancer cells cultured in acidic pH medium.

PPIs are among the most prescribed medications worldwide for peptic ulcers and gastroesophageal rerflux^33^^,^^34^. It has been reported that PPIs enhanced the effectiveness of anticancer agents^24–30^. Therefore, we investigated the effect of ESOM, a pharmacological PPI, on the activity of ROB and DOC in PC-3 cells. As shown in Figure 8(D), ESOM had a more potent inhibitory effect on PC-3 cells cultured in pH6.5 medium than that in pH7.4 medium. This finding is consistent with the result of an earlier study using melanoma cells^22^. Moreover, we found that ESOM enhanced the effects of ROB + DOC on PC-3 cells, and the effect of ESOM on the combination of ROB and DOC was significantly stronger in the cells in pH6.5 medium than that in pH7.4 medium (Figure 8(E)). These results indicate that PPIs may represent an effective approach for enhancing the efficacy of DOC and ROB in prostate cancer cells.

It is known that cancer cells have a characteristic microenvironment with reversed pH gradient resulted from an acidic extracellular pH and an alkaline intracellular pH. Recent studies have shown that PPIs decreased intracellular pH in cancer cells and enhanced the efficacy of paclitaxel (B, C). In the present study, we used the BCECF-AM probe to measure changes in cytosolic pH in PC-3 cells. As shown in Figure 8(F), treatment of PC-3 cells with ESOM reduced the fluorescence intensity indicating a decrease in intracellular pH in the cells. The combination of ESOM together with DOC and ROB also had a decreased fluorescence intensity. It is possible that the combined effect of ESOM with DOC and ROB on PC-3 cells was mediated at least in part by decreasing the cytosolic pH in the cells.

## 4. Conclusion

ROB and DOC in combination synergistically inhibited the growth of prostate cancer cells. The combination also strongly induced apoptosis, and suppressed cell migration, invasion and sphere formation. The strong combined effects of ROB and DOC on prostate cancer cells were associated with suppression of NF-κB, downregulation of Bcl-2 and upregulation of Bax. Moreover, the pharmacological PPI ESOM strongly enhanced the inhibitory effect of ROB and DOC on PC-3 cells. Results of our study indicate that combination of ROB and DOC together with PPIs may represent an effective approach for improving the treatment of prostate cancer patients.
